# Total Content and Composition of Phenolic Compounds from *Filipendula* Genus Plants and Their Potential Health-Promoting Properties

**DOI:** 10.3390/molecules29092013

**Published:** 2024-04-27

**Authors:** Ekaterina Sokolova, Tatiana Krol, Grigorii Adamov, Yulia Minyazeva, Dmitry Baleev, Nikolay Sidelnikov

**Affiliations:** All-Russian Scientific Research Institute of Medicinal and Aromatic Plants, Grina Street, 7, Moscow 117216, Russia; tatianakroll1@gmail.com (T.K.); grig.adamov@mail.ru (G.A.); miusly@yandex.ru (Y.M.); dbaleev@gmail.com (D.B.); vilarnii@mail.ru (N.S.)

**Keywords:** *Filipendula*, plant, phenolic compounds, anticoagulant activity, lipase, amylase

## Abstract

This current article was dedicated to the determination of the composition of phenolic compounds in extracts of four species of the genus *Filipendula* in order to establish a connection between the composition of polyphenols and biological effects. A chemical analysis revealed that the composition of the extracts studied depended both on the plant species and its part (leaf or flower) and on the extractant used. All four species of *Filipendula* were rich sources of phenolic compounds and contained hydrolyzable tannins, condensed tannins, phenolic acids and their derivatives, and flavonoids. The activities included data on those that are most important for creating functional foods with *Filipendula* plant components: the influence on blood coagulation measured by prothrombin and activated partial thromboplastin time, and on the activity of the digestive enzymes (pancreatic amylase and lipase). It was established that plant species, their parts, and extraction methods contribute meaningfully to biological activity. The most prominent result is as follows: the plant organ determines the selective inhibition of either amylase or lipase; thus, the anticoagulant activities of *F. camtschatica* and *F. stepposa* hold promise for health-promoting food formulations associated with general metabolic disorders.

## 1. Introduction

In the last 20 years, medicinal practitioners have pushed forward the concept of therapeutic effects via the gastrointestinal system [1]. As a result, a variety of foods labeled ‘functional’ have emerged. Currently, this is defined as ‘industrially processed or natural foods that, when regularly consumed within a diverse diet at efficacious levels, have potentially positive effects on health beyond basic nutrition’ [2]. Functional food is in the spotlight of extensive research covering the field of therapeutic plants. An unrivaled drive for the use of herbal preparations as prescription drugs in modern healthcare is charged by their enticing therapeutic potential and the general belief that they are safe. The concern about the safety attributed to plant-based therapeutics has already been raised, as has the level of study on the promised biological effects [3].

One of the approaches in the therapy of obesity and diabetes mellitus is the inhibition of the activity of the enzymes secreted in the lumen. Pancreatic lipase (EC 3.1.1.3; triacylglycerol acyl hydrolase) exerts its activity in conjunction with bile salts and co-lipase by releasing glycerol esters, 2-monoacylglycerols, glycerol, and free fatty acids from triglycerides [4]. The only approved anti-lipase drug is orlistat, so the search for new substances with this type of activity is ongoing [4]. In turn, pancreatic α-amylase (EC 3.2.1.1; α-1,4-glucan-4-glucanohydrolase) attracts no less attention than lipase because amylase catalyzes the first step in the digestion of starch, a main source of carbohydrates in the human diet [5]. Acarbose is an approved amylase inhibitor; however, it has been marked by some drawbacks [6].

Cardiovascular disease is yet another disorder whose prevalence worldwide is staggering and associated with obesity and diabetes mellitus [7,8]. Presently, the treatment of cardiovascular disease focuses on the application of oral anticoagulants. The reason is that oral anticoagulants demonstrated significant potency for the prevention and treatment of thrombosis and were associated with a decreased risk of potentially fatal bleeding. Licensed direct oral anticoagulants function via targeting thrombin or factor Xa and have a lower propensity for food and drug interactions than vitamin K antagonists [9].

Multiple examples of literature data report that plants are a rich source of compounds with potential inhibitory properties towards both pancreatic enzymes and coagulation pathway enzymes [10,11,12,13,14]. Currently, there are about 800 plant species known to exhibit antidiabetic properties [15]. For example, it has been found that an increase in galloyl groups in the structure of ellagitannins reduces the activity of amylase, while ellagitannins with β-galloyl groups in the C-1 glucose positions have a stronger inhibitory effect [16]. The inhibition of pancreatic lipase by phenolic compounds of plant extracts has also been demonstrated [17,18,19,20].

The genus *Filipendula* Mill belongs to the *Rosaceae* family, representatives of which grow in temperate and subarctic zones in the Northern Hemisphere [21]. The composition of *F. ulmaria* and *F. vulgaris* has been studied in more detail [22,23,24]. The aerial part of *F. ulmaria* (meadowsweet) is included in European Pharmacopoeia 10.5. [25,26]. Plants of the genus *Fillipendula* contain various classes of biologically active compounds, including salicylates (salicyaldehyde and methyl salicylate), phenolic acids, flavonoids and their glycosides, ellagitannins, and condensed tannins [21,22,23,27]. The genus *Filipendula* comprises several aromatic species that are cultivated worldwide due to their horticultural, aromatic, nutritional, and therapeutic value. If *Filipendula* plants are used in food products as flavoring agents, then in medicine, they are well-recognized for their ability to alleviate symptoms of cold, fever, arthritis, diarrhea, peptic ulcers, diphtheria, and cardiovascular disorders [28]. For all of the aforementioned properties, *Filipendula* plants have fluctuations in activity depending on plant species and organs.

The purpose of this article was to study the prospects of *Filipendula* plants in the functional food industry by performing a chemical analysis of the phenolic composition of the *F. stepposa*, *F. palmata*, *F. ulmaria*, and *F. camtschatica* extracts and their biological effects in vitro. According to the literature data, plants in the *Rosaceae* family contain a substantial amount of polyphenols, which, in the case of these plants, are better extracted with 80% acetone [29]. The aqueous extraction was performed under conditions previously established as optimal to produce food-grade extracts rich in phenols and with a minimum tannin fraction suitable for direct incorporation into food products [30]. The biological part of the investigation was composed of the anticoagulant properties and the ability to affect pancreatic lipase and α-amylase activity of phenolic-rich extracts. Two types of extractions were applied, aqueous and acetone extracts, and to add dimension to the study, two parts of every plant species were subjected to extraction: flowers and leaves.

## 2. Results and Discussion

Foods rich in phytochemicals and polyphenols are proposed to be able to protect against vascular dysfunction, promote vascular health, and reduce the risk of cardiovascular diseases both in vitro [13] and in vivo [14]. Additionally, substantial evidence indicates that regular dietary consumption of such foods favorably affects carbohydrate and lipid metabolism [31,32]. In our study, we focused on aqueous and acetone extracts. Since plant extracts are traditionally used as concoctions made of combinations of different ingredients [33], and due to the lack of efficacy of purified plant compounds used in drug discovery [34], we studied the phenolic composition of crude extracts of some officinal *Filipendula* plants and their biological effects on digestive enzymes and blood coagulation in vitro.

An analysis of the total content of phenolic compounds did not reveal a significant difference between aqueous and acetone extracts (see Table 1 below). However, a dependence on plant organs (*p* < 0.05) and plant species (*p* < 0.05) was observed. Flower extracts contained a higher amount of phenolic compounds than leaf extracts, which is most noticeable for the *F. palmata* extracts series. Aqueous flower extracts contained 380 mg GAE g^−1^ dry weight of phenolic content compared to 109 mg GAE g^−1^ dry weight in leaf extracts. Acetone extracts resulted in a smaller but still significant difference between organs up to 160 mg GAE g^−1^ dry weight of flower extracts above the value for the leaf ones. On average, the extraction of total phenolics was good in our experimental design because complete methanol extraction of aerial parts of *F. ulmaria* had previously been reported to be similar to our data and about 288 mg GAE g^−1^ [27].

At the same time, the results of the flavonoid contents showed the influence of both the species of plant and the extractant (acetone or water). Aqueous extraction yielded lower amounts of flavonoids. Extracts of *F. camtchatica* and *F. stepposa* contained fewer flavonoids than those of other plants. The dependence on the plant organ (leaf or flower) was generally not significant. In combination, the flavonoid constituents in the total phenolic pool of the acetone and water extracts signified that *Filipendula* species produced varied contents of phenolic compounds. The literature data for aqueous extracts (tea) from *F. ulmaria*, *F. camtschatica*, *F. denudata*, and *F. stepposa* also reported different phenolic profiles [35]. Additionally, our data suggested that acetone extracts from the *Filipendula* plants were richer in flavonoids than water ones.

We also studied the composition of phenolic compounds in the extracts and performed tentative identification of the phenolic compounds (Table 2). Various classes of phenolic compounds were present in the flowers and leaves of *F. camtschatica*, *F. palmata*, *F. stepposa*, and *F. ulmaria*: hydrolyzable tannins (galloyl glucose and ellagitannins), condensed tannins (dimers and trimers), phenolic acids and their derivatives, and flavonoids (catechins, derivatives of quercetin, and kaempferol). The acetone leaf extracts of *F. stepposa*, *F. ulmaria*, *F. camtschatica*, and *F. palmata* contained peaks of 18, 40, 35, and 29 phenolic compounds, respectively, while the acetone flower extracts of *F. stepposa*, *F. ulmaria*, *F. camtschatica*, and *F. palmata* had peaks of 46, 43, 40, and 47 phenolic compounds, respectively. Aqueous leaf extracts of *F. stepposa*, *F. ulmaria*, *F. camtschatica*, and *F. palmata* showed peaks of 26, 39, 35, and 36 phenolic compounds, respectively. The aqueous flower extracts of *F. stepposa*, *F. ulmaria*, *F. camtschatica*, and *F. palmata* had peaks of 48, 44, 49, and 40 phenolic compounds, respectively. The composition of the extracts studied depended both on the plant species and its part (leaf or flower) and on the extractant used.

**Figure 1:**
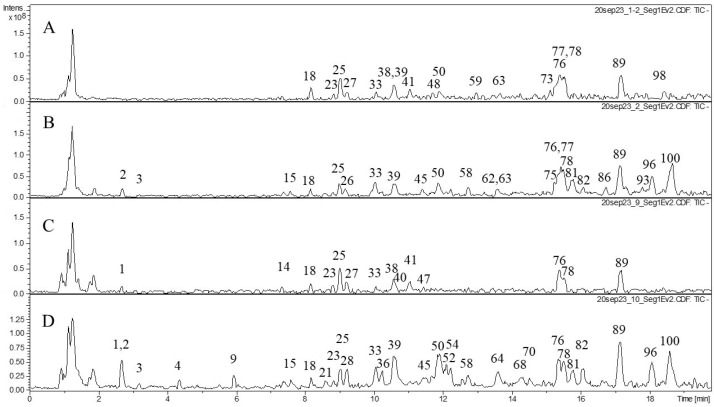
Profiles of HPLC data (TIC) on the phenolic compound composition of *F. stepposa* acetone and aqueous extracts. (**A**) acetone extraction of leaves; (**B**) acetone extraction of flowers; (**C**) aqueous extraction of leaves; (**D**) aqueous extraction of flowers.

**Figure 2:**
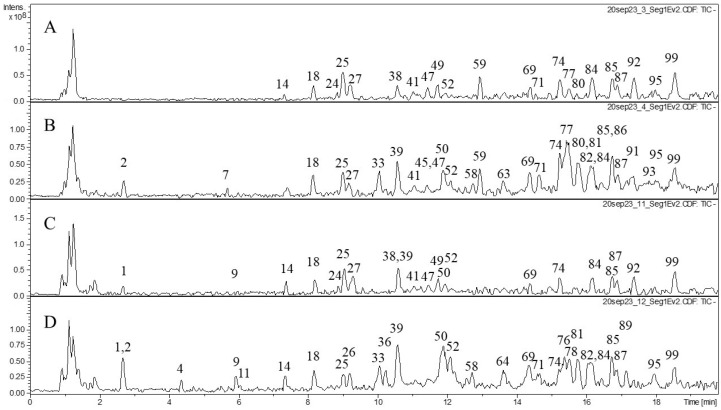
Profiles of HPLC data (TIC) on the phenolic compound composition of *F. palmata* acetone and aqueous extracts. (**A**) acetone extraction of leaves; (**B**) acetone extraction of flowers; (**C**) aqueous extraction of leaves; (**D**) aqueous extraction of flowers.

**Figure 3:**
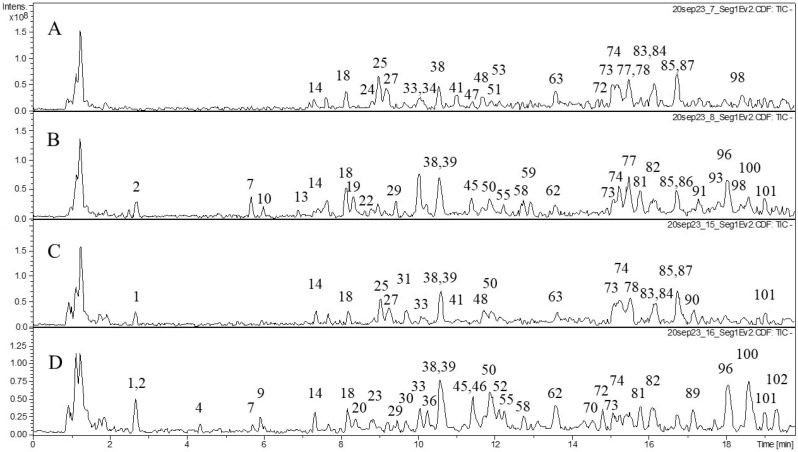
Profiles of HPLC data (TIC) on the phenolic compound composition of *F. ulmaria* acetone and aqueous extracts. (**A**) acetone extraction of leaves; (**B**) acetone extraction of flowers; (**C**) aqueous extraction of leaves; (**D**) aqueous extraction of flowers.

**Figure 4:**
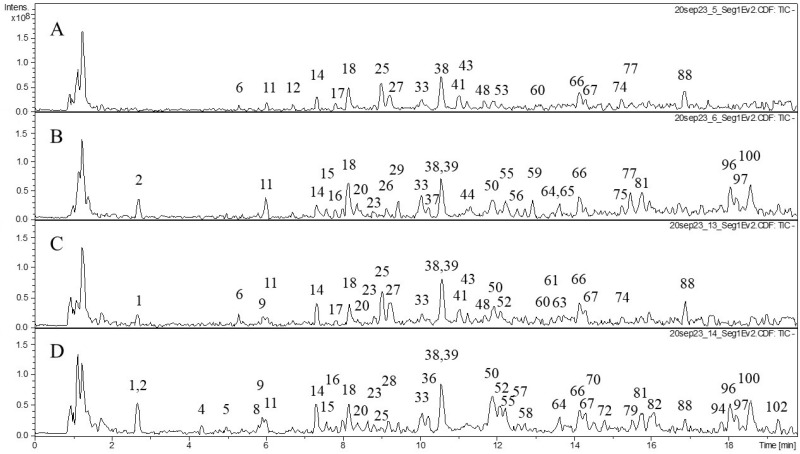
Profiles of HPLC data (TIC) on the phenolic compound composition of *F. camtschatica* acetone and aqueous extracts. (**A**) acetone extraction of leaves; (**B**) acetone extraction of flowers; (**C**) aqueous extraction of leaves; (**D**) aqueous extraction of flowers.

The composition of hydrolyzable tannins in flowers was more diverse compared to leaves (Table 2, Figure 1, Figure 2, Figure 3 and Figure 4). Monogalloyl hexoside (isomer 1) and digalloyl hexoside (isomer 3) are found only in meadowsweet flowers (*F. ulmaria*). The presence of monogalloylhexoside (isomer 2) was noted only in the flowers of *F. stepposa*. Digalloyl hexoside (isomer 1) was present in all aqueous flower extracts, and digalloyl hexoside (isomer 2) was present in the aqueous extracts of flowers and leaves except for the aqueous leaf extract of *F. stepposa*. Trigalloylhexoside (isomer 1) was present in the aqueous flower extracts of all species studied, as well as in the aqueous leaf extract of *F. camtschatica*. Its isomer is found in the acetone extracts of flowers, as well as the aqueous extracts of *F. ulmaria* and *F. camtschatica* flowers. Tetragalloyl glucose was present in the acetone extracts of *F. ulmaria* and *F. palmata*, as well as in the acetone extract of *F. stepposa* flowers. Pentagalloyl glucose was found in the acetone flower extracts of *F. stepposa*, *F. ulmaria*, and *F. palmata*, as well as in the aqueous extract of *F. stepposa*.

Rugosin E (isomer 1) was identified only in the flower acetone extracts of *F. camtschatica*, *F. palmata*, and *F. stepposa*; rugosin E (isomer 2) was found exclusively in the acetone extracts of all *Filipendula* species studied, and tellimagrandin II was present in all studied flowers, regardless of the extractant used. Flowers of *F. stepposa*, *F. ulmaria*, and *F. palmata* were also marked by the accumulation of rugosin A. This compound was absent only in the acetone leaf extract of *F. camtschatica*. Rugosin D was present in acetate extracts of *F. ulmaria* and *F. palmata*, as well as in acetone flower extracts of *F. stepposa* and *F. camtschatica*. Tellimagrandin I (isomer 1) was detected in almost all samples, except for the aqueous leaf extract of *F. stepposa* and the acetone leaf extract of *F. ulmaria*. Tellimagrandin I (isomer 2) was not detected in the acetone extract of *F. ulmaria* leaves. Tellimagrandin (isomer 3) was identified in the flowers of *F. stepposa* and *F. camtschatica*, as well as in the aqueous flower extract of *F. palmata*.

Catechins and condensed tannins were present mainly in the leaves. No condensed tannins were detected in acetone and aqueous extracts from *F. ulmaria* and *F. camschatica* flowers, as well as in the aqueous extract of *F. palmata* flowers.

A total of 16 quercetin derivatives were identified, most of which were present in the leaves and flowers of *F. palmata* and *F. ulmaria*.

Twenty-three compounds were attributed to phenolic acids and their derivatives: caftaric acid, caffeoyl-threonic acid isomers, coumaroyl threonic acid isomers, dihydroxybenzoic acid glucoside isomers, dihydrocaffeic acid glucuronide isomers, caffeic acid hexoside isomers, and coumaroyl hexoside. At the same time, only compounds 4 and 3 from this group were identified in the acetone leaf extracts of *F. stepposa* and *F. palmata*, respectively.

Of the salicylic derivatives, only salicylic acid hexoside and isosalicin were observed. At the same time, isosalicin accumulated only in the flowers of *F. camtschatica* and *F. stepposa*. Salicylic acid hexoside was not detected in *F. stepposa* but was present in the acetone extracts of flowers of the other species, as well as in the acetone leaf extract of *F. ulmaria* and the aqueous flower extract of *F. palmata*.

Valoneic acid dilactone was detected only in the aqueous flower extracts of *F. camtschatica*, *F. palmata*, and *F. stepposa* and in the aqueous leaf extract of *F. palmata*

The coagulation cascade is an intricate system of clotting factors where inactive precursors, after activation through proteolysis, catalyze the formation of the next sequential factor. This cascade occurs via one of two pathways: (1) the extrinsic, or tissue factor (measured by prothrombin time, PT), and (2) the intrinsic, or contact pathway (measured by activated partial thromboplastin time, APTT). Elevated coagulation is generally associated with cardiovascular disorders such as coronary artery disease, hypertension, atherosclerosis, etc. [46].

**Figure 5:**
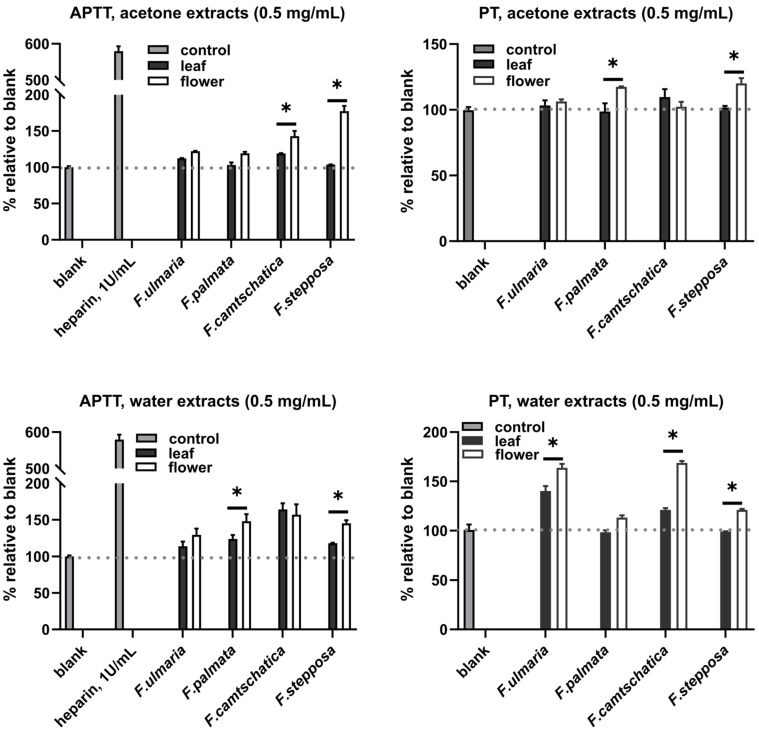
Anticoagulant activity of *F. stepposa*, *F. palmata*, *F. ulmaria*, and *F. camtschatica* of aqueous and acetone extracts measured by the APTT and PT tests. * *p* < 0.05.

Over the years, medicinal herbs for cardiovascular disorders have proven to be prospective to serve as oral anticoagulants, as revealed by their influence on laboratory data on several cardiovascular disease risk factors. Thus, there have been a great deal of attempts to relate studies on medicinal herbs to cardiovascular disease treatments [3]. In this study, aqueous and acetone extracts of four *Filipendula* plants were investigated to determine their effects on red blood cell hemolysis and coagulation. In general, no hemolysis was induced by the extracts tested, but they had an effect on coagulation time. Among the acetone extracts of *Filipendula* plants, only the flower ones exhibited tangible prolonging action on coagulation. In the APTT assay, flower extracts of *F. camtschatica* and *F. stepposa* (increase by 50 and 100%, respectively) showed a significant and pronounced effect, while a drastically less potent but still significant elongation of the PT time was observed by extracts of *F. palmata* and *F. stepposa* (increase by about 10–15%) (Figure 5). The aqueous extracts were active in equal measure, prolonging both the APTT and PT, but the tendency for stronger effects by flower extracts was preserved. Out of all the data as regards water extracts, *F. camtschatica* extracts were the most active (prolonging coagulation by approximately 50%), which was fair even for leaf extracts in the APTT (Figure 5). Overall, it can be concluded that the extracts from *F. camtschatica* were noticeably more active anticoagulants.

Our present results are especially important since few studies have been carried out to determine the direct effect of plant extracts on blood parameters such as hemolysis and coagulation, and even less so for widely used medicinal plants of the genus *Filipendula*. According to the literature, *F. ulmaria* possessed anticoagulant activity because of a heparin-like compound [28,47]; however, the chemical characterization of that compound has not been described. The intensity of the anticoagulant action in our experiments was exceptionally lower than that of heparin, and in addition, *F. ulmaria* extracts were weaker than *F. camtschatica* ones. The phytochemical analysis of *F. camtschatica* extracts differentiates them from other studied *Filipendula* plants by the distinguished presence of dihydrocaffeic acid 3-*O*-glucuronide (**11**), bergenin or its isomer (**12**), and isomers galloyl-caffeoyl-threonic acid (**66**, **67**) compounds (Figure 4, Table 2). Whether those phytochemicals could be responsible for the anticoagulant effect is a matter of further research because, previously, there have been only a few studies dedicated to anticoagulant action in plants. Some explanations were tethered in the field of phenolic substances—for example, the inhibitory potential of coumarin [48] or phenolic-compound-enriched extracts from black chokeberry and grape seeds on coagulation factors [49]—whereas another study focused on macromolecular polysaccharide–polyphenolic conjugates as anticoagulant-rendering substances [50]. Here, it seems pertinent to mention that isosalicin derivatives of maltose were powerful anticoagulant agents in in vitro assays [51,52,53]. In our research, isosalicin (**15**) (Figure 1 and Figure 4, Table 2) was found only in aqueous extracts of *F. camtschatica* and *F. stepposa* flowers, which have been among the strongest test extracts in the anticoagulant experiments (Figure 5). It is important to raise awareness of the multiple biological effects of therapeutic herbs to make the most of their potential and acknowledge possible drawbacks depending on an individual’s state of health.

**Figure 6:**
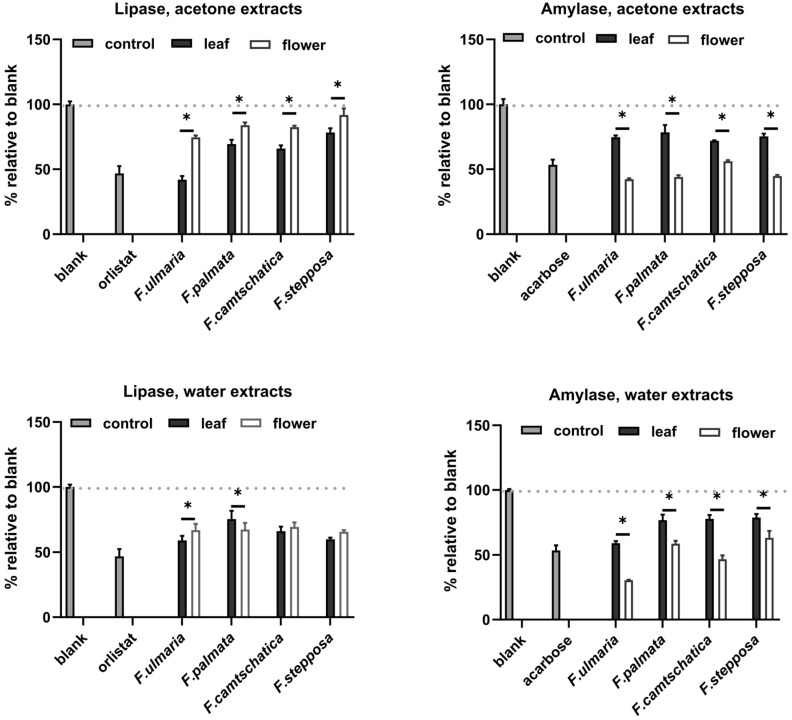
Influence of aqueous and acetone extracts of *F. stepposa*, *F. palmata*, *F. ulmaria*, and *F. camtschatica* flower and leaf extracts on the activity of the digestive enzymes amylase and lipase. * *p* < 0.05.

Our results demonstrated that all extracts tested had the ability to inhibit pancreatic lipase and amylase activity. In both tests, the *Filipendula* plant extracts were less effective than the positive control for each enzyme. For amylase inhibition, the difference in the intensity of inhibition between different organs is pronounced (on average, 50% inhibition by flowers versus 25% inhibition by leaf extracts). *F. ulmaria* extracts showed a more prominent amylase inhibitory potential than the rest of the test plants. Previously, teas of *F. camtschatica*, *F. denudate*, *F. stepposa*, and *F. ulmaria* were studied for their pancreatic amylase inhibitory activity [35]. Out of all the aforementioned species, *F. denudata* tea had the highest inhibitory activity and *F. camtschatica* had the weakest. It should be noted that the amylase activity test made by Olennikov and co-authors [35] was different. The amylase assay with the CNP substrate used in our article was a variant of conventional clinical amylase assays. The amylase inhibitory effect of acetone extracts of *Filipendula* plants has not been an object of interest before, and this present work revealed that their inhibitory level was not particularly different from that of aqueous extracts (Figure 6). The literature reports suggested that phenolic compounds with evident anti-amylase activity belong to ellagitannin, especially rugosin D, which was more active than acarbose [35,54,55]. Rugosin A was also capable of inhibiting amylase [56]. Phytochemical analysis in our study demonstrated several ellagitannins: rugosin B (**52**, **57**), rugosin E (**75**, **77**), rugosin A (**82**), and rugosin D (**91**) (Table 2). However, only rugosin A was present in all flower extracts; the other rugosins were scattered among plant species, extracts, and organs, except for rugosin E (**77**), detected in all acetone extracts (Table 2, Figure 1, Figure 2, Figure 3 and Figure 4). Thus, the reason why flower extracts of *F. ulmaria* were the best inhibitors is a matter of further research, but rugosin A could be responsible for the general direction of amylase inhibition by *F. camtschatica*, *F. denudate*, *F. stepposa*, and *F. ulmaria* flower extracts.

With regard to lipase, this trend can be seen only for acetone extracts from *F. ulmaria* (inhibition by 57%); on average, the effects of all other plants showed a similar intensity in the range of 10–40% without significant dependence on the extractant, plant organ, or plant species, except for acetone extracts from *F. ulmaria* (Figure 6). *Filipendula* plants have not been extensively studied regarding their effect on lipase, but one study determined that 3-*O*-caffeoyl-4-*O*-galloyl-L-threonic acid, a phytochemical isolated from *F. camtschatica*, inhibited pancreatic lipase with a half maximal concentration of 26 μM [57,58]. The current results indeed showed the presence of galloyl-caffeoyl-threonic acid (**66**, **67**) in *F. camtschatica* extracts with no dependence on the extractant or the plant part. It had also been previously observed that the presence of a galloyl group in the structure of phenolic compounds had a positive effect on increasing the inhibitory effect on lipase [4]. Thus, tannic acid and pentagalloyl glucose are more effective inhibitors of pancreatic lipase than, for example, chlorogenic and protocatechuic acids [59]. However, it was impossible to attribute anti-lipase action solely to galloyl-caffeoyl-threonic acid because it was absent in other *Filipendula* extracts and had no visible detrimental effect on the lipase-inhibiting properties (Figure 6, Table 2). One of the tendencies in our results concerned catechins and condensed tannins, which were present predominantly in the leaves of the tested *Filipendula* plants (Table 2). The lipase-inhibiting properties were allocated to condensed tannins, which comprised a significant part of the extract of *Araucaria angustifolia* [60]. In general, out of the four *Filipendula* plants in our case, *F. ulmaria* acetone leaf extracts were the strongest inhibitors of lipase, suggesting the presence of some other important phytochemicals. 

## 3. Materials and Methods

The voucher specimens were deposited in the Voucher Fund, All-Russian Scientific Research Institute of Medicinal and Aromatic Plants, Moscow, Russia: (*Filipendula camtschatica* (Pall.) Maxim. Specimen No. VF 008.23 collected on 25 July 2023, *F. ulmaria* (L.) Maxim. Specimen No. VF 009.23 collected on 12 Jul 2023, *F. stepposa* Juz.—synonym, priority name—*Filipendula ulmaria* subsp. picbaueri (Podp.) Smejkal Specimen No. VF 011.23 collected on 12 July 2023, and *F. palmata* (Pall.) Maxim. Specimen No. VF 012.23 collected on 25 July 2023). The collected leaves were stored at −80 °C and freeze-dried for 72 h (LABCONCO^®^ FreeZone 2.5 L, Kansas City, MO, USA).

### 3.1. Acetone and Aqueous Extractions

*Extraction with acetone* was performed as previously described [29,61]. Sample material (leaves or flowers, 20.0 mg) was ground using a vibrating mill at 20 Hz (MM 400, Rersch, Haan, Germany) for 2–4 min, followed by maceration in 1 mL of 80% acetone and incubation under constant stirring for 60 min. The extract was separated by centrifugation for 15 min at 14,000 rpm. The extraction was repeated two more times. The solvent for the combined extract was entirely removed under vacuum at 40 °C (CentriVap DNA Concentrator, LABCONCO^®^, USA) to obtain the dry extracts.

*Extraction with water* (infusion, tea) was made according to the recommendations of *F. ulmaria* by N. Harbourne and co-authors [30]. Briefly, a finely powdered sample (20 mg) was steeped in MilliQ water (1 mL) and incubated at 92.5 °C for 15 min, and then immediately cooled on ice and centrifuged for 15 min at 14,000× *g* (Centrifuge 5430 R, Eppendorf^®^, Hamburg, Germany). The supernatants were collected and freeze-dried for 72 h. 

### 3.2. Total Flavonoid Content

Flavonoid content was measured based on the chelate formation with AlCl_3_ in acetate buffer described elsewhere [62,63]. Briefly, the wells of a 96-well microplate contained 140 μL of H_2_O, 5 μL of 10% AlCl_3_, 5 μL of 1 M sodium acetate, and 100 μL of 1 mg mL^−1^ sample, which were added to the wells in triplicate and incubated for 30 min at room temperature. Quercetin was used as a positive control, and the blank was the same as the assay wells, but 10% AlCl_3_ was substituted with the same amount of water. The absorbance was read at a wavelength of 415 nm (SPECTROstar Nano, BMG LABTECH^®^, Ortenberg, Germany), and the results calculated the mg Quercetin equivalent per g of dry weight. Stock quercetin solution was prepared by dissolving in EtOH; the reference solutions were serial dilutions from 6.25 to 200 μg mL^−1^.

### 3.3. Phenolic Content 

The total phenolic content in the extracts and teas of *Filipendula* was assessed using the Folin–Ciocalteu assay [64] in 96-well microplates [65]. Briefly, 10 μL of 1 mg mL^−1^ samples was mixed with 100 μL of Folin–Ciocalteu reagent (10-fold diluted) and 80 μL of 1 M sodium carbonate, and the plate was shaken for 20 s. After 20 min incubation in the dark at room temperature, the absorbance was measured at λ = 630 nm. Total phenolic content was expressed as mg gallic acid equivalent per gram of dry weight (mg GAE g^−1^). 

### 3.4. HPLC Analysis of Phenolic Contents

Analysis of phenolic contents was performed by means of a chromatography system equipped with a tandem mass spectrometry detector and an electrospray ionization (ESI) system (LCMS-8045, Shimadzu, Tokyo, Japan). Primary data were processed using LabSolutions (Ver. 5.3) (Shimadzu Corporation, Tokyo, Japan). Mass spectrometric detection of compounds was carried out in total ion current (TIC), gas flow-3 L min^−1^, gas temperature 300 °C, nebulizer-3 L min^−1^, in negative and positive modes in the range of 100–2000 *m/z*.

For chromatographic separation, an ACQUITY UPLC^®^ BEH Phenyl column (100 × 2.1 mm, 1.7 µm, Waters, Wexford, Ireland) was used in gradient mode. Mobile phase A—0.1% formic acid (Sigma-Aldrich, St Louis, MO, USA) in water, mobile phase B—100% acetonitrile (HPLC grade, MACRON, Gliwice, Poland). Mobile phase flow rate: 0.25 mL min^−1^. The column thermostat temperature was 40 °C. Elution program: 0.0–1.0 min, 1% B; 1.0–25.0 min, 1–30% B; 25.0–30.0 min, 30–40 B; 30.0–35.0 min, 40% B; 35.0–36.0 min, 40–90% B; 36.0–38.0 min, 90% B; 38.0–39.0 min, 90–1% B; 39.0–39.5 min, 1–1% B.

Identification of compounds was made using mass spectrometric data by comparison with the characteristics of known compounds from the open database The Human Metabolome Database (HMDB Version 5.0), as well as with published data from other researchers [21,22,23,35,36,37,39,42,43].

### 3.5. Blood Collection and Plasma Preparation

Human blood was drawn from the antecubital vein of normal healthy volunteers in vacuumed plastic tubes VacPlus^®^ (“Hebei Xinle Sci & Tech”, Xinle county, Shijiazhuang, Hebei province, China) with 3.8% sodium citrate. All volunteers gave written informed consent for the blood collection and use at the All-Russian Scientific Research Institute of Medicinal and Aromatic Plants, Russia. Blood plasma for anticoagulant assays was obtained by centrifuging for 15 min at 1200× *g*.

### 3.6. Prothrombin Time

Samples (acetone and aqueous extracts at a final concentration of 0.5 mg mL^−1^) and heparin (1 U mL^−1^ final concentration) were incubated for 1 min at 37 °C with 28.5 μL of human plasma, followed by the addition of 0.06 mL of a solution of a thromboplastin calcium mixture (RPA “Renam”, Moscow, Russia) preheated at 37 °C. The clotting time was registered.

### 3.7. Activated Partial Thromboplastin Time

The investigated samples (acetone and aqueous extracts at a final concentration of 0.5 mg mL^−1^) and heparin (1 U mL^−1^ final concentration) and 28.5 μL of human plasma were incubated for 3 min at 37 °C with 28.5 μL of the aPTT reagent (cephalin kaolin suspension) (RPA “Renam”, Russia). The addition of 28.5 μL of CaCl_2_ at 0.025 M initiated the coagulation process, and the timer was started.

### 3.8. Lipase Activity

The lipase activity was assessed based on a modified protocol developed by Panteghini, Bonora, and Pagani [66]. The acetone extracts were diluted in DMSO to a final concentration of 200 μg mL^−1^ final concentration in the assay solution and the tea solutions (final 200 μg mL^−1^). Orlitstat was used as a positive control (116 ng mL^−1^) and the activity of lipase without orlistat or the investigated samples was the negative control (blank). Prior to the reaction, all reagents were heated to 37 °C. The assay was carried out in a 96-microplate, and the wells were filled sequentially with 2 μL of lipase solution (60 U mL^−1^) (according to European Pharmacopeia) with porcine pancreatic lipase in deionized water as a source of lipase, 90 μL of Solution 1 (40 mM, pH 8.0 Tris buffer, 1 mg L^−1^ colipase, 6.4 mM deoxycholate sodium salt, 3.4 mM taurodeoxycholate sodium salt, 0.09% sodium aside, 7.4 mM calcium chloride) (Vector Best, Novosibirsk, Russia), and 5 μL of sample solution. After incubation for 2 min under shaking at 37 °C, 18 μL of Solution 2 containing substrate 1,2-*O*-dilauryl-rac-glycero-3-glutaric acid-(6-methyl resoruphin) ester (DGGR) (7.5 mM, pH 4.0 potassium sodium tartrate buffer, and 1 mM DGGR) (Vector Best, Novosibirsk, Russia) was added to initiate the development of the colored reaction at λ = 580 nm. The number of measurements was three to four times.

### 3.9. Amylase Activity

The effect of the plant extracts on pancreatic amylase activity was measured by the direct colorimetric method for specific determination of the pancreatic isoenzyme described elsewhere [67]. Salivary amylase was blocked with anti-human salivary amylase monoclonal antibodies. Intact pancreatic amylase splits substrate 2-chloro-4-nitrophenol-oligosaccharide, yielding free 2-chloro-4-nitrophenyl (CNP), which is monitored at 405 nm. The concertation range of the acetone extracts in DMSO and the aqueous extracts in MilliQ. Acarbose was used as a positive control (0.24 μg mL^−1^) and water was used as a negative control. Prior to the reaction, all the reagents were heated to 37 °C. The assay was performed in a 96-microplate, 2.5 μL of amylase solution (50 U mL^−1^), 95 μL of 50 mmol/L MES buffer (pH 6.0) containing anti-human salivary amylase monoclonal antibodies and 0.05% aside sodium (Vector Best, Novosibirsk, Russia), and 5 μL of sample solution (200 μg mL^−1^ final concentration). After intensive shaking and incubation for 5 min at 37 °C, a solution containing the substrate (23 μL, 20 mmol mL^−1^) in MES buffer (50 mmol mL^−1^, pH 6.0) and 0.09% aside sodium (Vector Best, Novosibirsk, Russia) was added. The intensity of color development at λ = 405 nm was monitored. The measurements were performed in triplicate. 

### 3.10. Statistical Analysis 

The data are expressed as mean ± standard deviation. A two-way analysis of variance (ANOVA) followed by Sidak’s multiple comparisons test for group means was applied to analyze the significant difference. The results were considered statistically significant if *p* < 0.05. 

## 4. Conclusions

In summary, this current article was dedicated to the determination of the composition of phenolic compounds in extracts of four species of the genus *Filipendula* in order to establish the connection between the composition of polyphenols and biological effects. The chemical analysis revealed that the composition of the extracts studied depended both on the plant species and its part (leaf or flower) and on the extractant used. All four species of *Filipendula* were rich sources of phenolic compounds and contained hydrolyzable tannins, condensed tannins, phenolic acids and their derivatives, and flavonoids. The activities included data on those that are most important for creating functional foods with *Filipentdula* plant components: the influence of prothrombin and activated partial thromboplastin time on blood coagulation, and inhibition of the digestive enzymes pancreatic amylase and lipase. It was established that plant species, parts, and extraction methods contributed meaningfully to biological activity. The most prominent results are as follows: plant organs determine the selective inhibition of either amylase or lipase effects; thus, the anticoagulant activities of *F. camtschatica* and *F. stepposa* hold promise for health-promoting food formulations associated with general metabolic disorders.

## Figures and Tables

**Table 1:** Qualitative contents of total phenolic compounds of *F. stepposa*, *F. palmata*, *F. ulmaria*, and *F. camtschatica* acetone and aqueous extracts.

	Yield (mg/g)				Total Polyphenol Contents (Gallic Acid eq, mg/g d.w.)				Flavonoid Contents (Quercetin eq, mg/g d.w.)			
Plant Species	Water		Acetone		Water		Acetone		Water		Acetone	
	Leaf	Flower	Leaf	Flower	Leaf	Flower	Leaf	Flower	Leaf	Flower	Leaf	Flower
*F. ulmaria*	301.2	350.1	450.8	477.3	202.0 ± 4.1	289.4 ± 7.3	216.6 ± 8.7	320.5 ± 5.0	34.4 ± 4.3	29.9 ± 2.0	41.9 ± 0.6	42.3 ± 1.9
*F. palmata*	217.1	377.9	326.3	513.7	109.3 ± 5.2	381.1 ± 4.7	209.0 ± 2.7	371.1 ± 13.9	31.3 ± 6.1	38.2 ± 5.6	53.3 ± 0.9	34.3 ± 4.5
*F. camtschatica*	305.9	368.3	372.3	483.7	188.1 ± 9.5	248.7 ± 10.5	243.3 ± 7.3	260.8 ± 4.7	7.2 ± 0.1	16.7 ± 1.3	23.3 ± 4.3	19.6 ± 1.5
*F. stepposa*	200.7	448.4	330.4	531.2	122.3 ± 4.3	215.9 ± 13.3	159.0 ± 4.2	289.3 ± 10.9	15.5 ± 1.7	20.4 ± 1.3	33.6 ± 2.9	22.5 ± 3.7

**Table 2:** HPLC-MS characterization of phenolic compounds of *F. stepposa*, *F. palmata*, *F. ulmaria*, and *F. camtschatica* acetone and aqueous extracts.

No.	Compound (Ref.)	RT, min	Mass Spectrum				*F. stepposa*				*F. ulmaria*				*F. camtsch.*				*F. palmata*			
							Ac ^1^		Aq ^1^		Ac		Aq		Ac		Aq		Ac		Aq	
			[M − H]^−^/[2M − H]^−^ (*m*/*z*)	[M − 2H]^2−^ (*m*/*z*)	[M+H]^+^ (*m*/*z*)	Fragm. (*m*/*z*)	L ^1^	F	L	F	L	F	L	F	L	F	L	F	L	F	L	F
Phenolic acids and their derivatives																						
1	Gallic acid [23,36]	2.65	169/339	-	-	-																
5	Dihydrocaffeic acid -glucuronide, isomer 1; HMDB ^1^	4.96	357/715	-	-	-																
7	Dihydroxybenzoic acid-glucoside, isomer 1; HMDB	5.65	315/-	-	-	-																
8	Dihydrocaffeic acid -glucuronide, isomer 2, HMDB	5.78	357/715	-	-	-																
11	Dihydrocaffeic acid -glucuronide, isomer 3, HMDB	6.00	357/715	-	-	-																
12	Bergenin, HMDB	6.67	327/655	-	-	-																
13	Dihydroxybenzoic acid-glucoside, isomer 2, HMDB	6.88	315/-	-	-	-																
14	Caffeoyl-threonic acid, isomer 1 [22,23]	7.31	297/595	-	-	-																
15	Isosalicin, isomer 1 [23]	7.57	285/-	-	-	-																
17	Caffeoyl-threonic acid, isomer 2 [22,23]	7.79	297/-	-	-	-																
18	Caffeoyl-threonic acid, isomer 3 [22,23]	8.14	297/595	-	-	-																
19	Isosalicin, isomer 2 [23]	8.31	285/-	-	-	-																
20	Dihydrocaffeic acid -glucuronide, isomer 4, HMDB	8.37	357/-	-	-	-																
21	Caffeoyl pentoside, isomer [37]	8.56	311/-	-	313	181																
22	Salicylic acid hexoside [23,38]	8.59	299/-	-	-	-																
23	Dihydrocaffeic acid -glucuronide, isomer 5, HMDB	8.78	357/-	-	359	181																
28	Coumaroyltreonic acid, isomer 1 [23]	9.20	281/-	-	-	-																
29	Caffeic acid hexoside, isomer 1, HMDB; [39]	9.40	341/-	-	343	181																
30	Caffeic acid hexoside, isomer 2, HMDB; [39]	9.67	341/-	-	-	-																
32	Coumaroyltreonic acid, isomer 2 [23]	9.91	281/-	-	283	165																
33	Coumaroyltreonic acid, isomer 3 [23]	10.02	281/563	-	-	165																
37	Caffeic acid hexoside, isomer 3, HMDB; [39]	10.21	341/-	-	-	-																
38	caffeoyl-threonic acid, isomer 4 [22,23]	10.55	297/595	-	299/597	181																
40	Caffeic acid hexoside, isomer 4, HMDB; [39]	10.65	341/-	-	343	181																
43	Caffeoyl-threonic acid, isomer 5 [23]	11.20	297/-	-	-	-																
45	Coumaroyl hexoside [39]	11.37	325/-	-	327	165																
46	Caffeic acid hexoside, isomer 5, HMDB; [39]	11.37	341/-	-	-	-																
58	Coumaroyltreonic acid, isomer 4 [23]	12.72	281/-	-	-	165																
70	Ellagic acid [21]	14.50	301/-	-	-	-																
15	Isosalicin, isomer 1 [23]	7.57	285/-	-	-	-																
Hydrolyzable tannins																						
2	Monogalloylhexoside, isomer 1 [23]	2.67	331/663	-	-	-																
3	Monogalloylhexoside, isomer 2 [23]	3.17	331/-	-	-	-																
4	Digalloylhexoside, isomer 1, [23]	4.32	483/-	-	-	-																
6	Galloyl-threonic acid [23]	5.28	287/575	-	-	-																
9	Digalloylhexoside, isomer 2 [23]	5.88	483/-	-	-	-																
10	Monogalloylhexoside, isomer 3 [23]	5.97	331/-	-	-	-																
26	Digalloylhexoside, isomer 3 [23]	9.13	483/-	-	-	-																
36	Trigalloylhexoside, isomer 1 [23]	10.19	635/-	-	-	-																
39	Tellimagrandin I, isomer 1 [21,23,35]	10.55	785/1571	392	-	-																
48	Ellagic acid-pentoside [37,40]	11.64	433/-	-	-	-																
50	Tellimagrandin I, isomer 2 [35,36]	11.86	785/1571	392	787	-																
52	Rugosin B, isomer 1 [23,35,36]	12.09	953/1907	476	-	-																
55	Trigalloylhexoside, isomer 2 [23]	12.23	635/-	-	-	-																
57	Rugosin B, isomer 2 [35]	12.53	953/-	476	-	-																
64	Tellimagrandin I, isomer 3 [23]	13.59	785/-	-	-	-																
66	Galloyl-caffeoyl-threonic acid, isomer 1 [23,36]	14.11	449/899	-	-	181																
67	Galloyl-caffeoyl-threonic acid, isomer 2 [23]	14.27	449/-	-	-	181																
68	Galloyl-bis-HHDP-glucose, isomer 1 [23]	14.3	935/-	467	-	-																
75	Rugosin E, isomer 1, [21,23,35,36]	15.24	1722/-	860	-	-																
77	Rugosin E, isomer 2 [35]	15.43	1722/-	860	-	-																
79	Galloyl-bis-HHDP-glucose, isomer 2 [23]	15.48	935/-	467	-	-																
80	Tetragalloylglucose [23]	15.7	787/-	-	-	-																
81	Tellimagrandin II [21,23,36]	15.75	937/1875	468	-	-																
82	Rugosin A [23,36]	16.05	1105/-	552	-	-																
91	Rugosin D [21,36]	17.31	1874/-	936	-	-																
93	Pentagalloylglucose [23]	17.78	939/-	469	-	-																
94	Digalloyl-caffeoyl-threonic acid [23]	17.8	601/-	-	-	-																
98	Bicornin [23]	18.36	1087/-	543	-	-																
Flavanols and proanthocyanidins																						
24	(Epi)catechin-(epi)catechin, isomer 1 [23]	8.81	577/-	-	579	291; 289																
25	Catechin [23,36]	8.98	289/579	-	291	-																
27	(Epi)catechin-(epi)catechin, isomer 2 [22]	9.2	577/-	-	579	291; 289																
34	Procyanidin trimer, isomer 1 [23,37]	10.04	865/-	-	-	-																
35	Procyanidin trimer, isomer 2 [23]	10.14	865/-	-	867	579; 283																
41	(Epi)afzelechin-(epi)catechin, isomer [37,41]	11.00	561/-	-	563	273																
47	(Epi)catechin [23]	11.44	289/-	-	291	-																
51	(Epi)afzelechin-(epi)catechin-(epi)catechin, isomer [37]	11.89	849/-	-	851	579; 273																
53	(Epi)catechin-(epi)catechin, isomer 3 [22]	12.09	577-	-	-	-																
60	(Epi)afzelechin-(epi)afzelechin-(epi)catechin, isomer 1 [42]	13.16	833/-	-	835	563; 273																
61	(Epi)afzelechin-(epi)afzelechin-(epi)catechin, isomer 2 [42]	13.39	833/-	-	835	563; 291; 273																
63	(Epi)catechin-(epi)catechin-gallate, isomer [37]	13.56	729/-	-	731	291																
Kaempferol derivatives																						
42	Kaempferol-rhamnoside [35]	11.14	431/-	-	-	-																
89	Kaempferol-glucuronide, HMDB	17.11	461/923	-	463	287																
90	Kaempferol-glucoside [35,43]	17.14	447/-	-	449	287																
97	Kaempherol-acetyl-hexoside, HMDB	18.17	489/-	-	-	287																
100	Kaempferol derivative [23,42]	18.56	447/895	-	449	317; 287																
Quercetin derivatives																						
69	Quercetin-galloylglucoside, isomer 1 [42]	14.35	615/-	-	617	303																
71	Quercetin-galloylglucoside, isomer 2 [42]	14.57	615/-	-	617	303																
72	Quercetin-rutinoside, isomer 1 [42]	14.77	609/-	-	611	303																
73	Quercetin-rutinoside, isomer 2 [42,43]	15.02	609/1219	-	611	303																
74	Quercetin-glucoside, isomer 1 [35,42,43]	15.21	463/927	-	465	303																
76	Quercetin-glucuronide, isomer [35]	15.33	477/955	-	479	303																
78	Quercetin-glucoside, isomer 2 [43]	15.48	463/-	-	465	303																
83	Quercetin-galloyldihexoside, isomer [23]	16.1	761/-	-	-	303																
84	Quercetin-pentoside, isomer 1 [35]	16.13	433/867	-	435	303																
85	Quercetin-pentoside, isomer 2 [35]	16.7	433/-	-	435	303																
87	Quercetin-acetyl-glucoside, isomer, HMDB	16.77	505/-	-	-	-																
88	Quercetin-3-*O*-malonylglucoside, isomer [44]	16.86	549/1099	-	551	303																
92	Quercetin-rhamnoside, isomer [35]	17.35	447/-	-	449	303																
95	Quercetin-*O*-(*O*-galloyl)-pentoside, isomer [45]	17.92	585/-	-	587	303																
102	Quercetin-3-*O*-(5″-*O*-malonyl)-arabinofuranoside, isomer [44]	19.30	519/-	-	521	303																
103	Quercetin-3-*O*-(4″-*O*-malonyl)-rhamnoside, isomer [44]	19.87	533/1067	-	535	303																
Other																						
54	Valoneic acid dilactone, HMDB	12.20	469/-	-	-	-																
Unknown phenolic compounds																						
16	Unknown	7.65	633/-	-	-	-																
31	Unknown	9.69	439/879	-	439	-																
44	Unknown	11.29	447/-	-	449	-																
49	Unknown	11.71	431/-	-	-	-																
56	Unknown	12.50	491/-	-	-	-																
59	Unknown	12.92	491/-	-	-	-																
62	Unknown	13.54	377/-	-	-	-																
65	Unknown	13.71	461/-	-	-	-																
86	Unknown	16.71	1329/-	-	-	-																
96	Unknown	18.02	463/927	-	465	303; 287																
99	Unknown	18.53	475/-	-	-	-																
101	Unknown	18.98	601/-	-	-	317																

^1^ Abbreviations used: Ac—acetone extraction, Aq—aqueous extraction, L—leaf, F—flower, HMDB Version 5.0—the Human Metabolome Database. 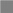
—designates ‘detectable amounts’

## Data Availability

Data are contained within the article.

## References

[1] Gupta H., Garg S. (2020). Obesity and overweight—Their impact on individual and corporate health. J. Public. Health.

[2] Granato D., Barba F.J., Bursać Kovačević D., Lorenzo J.M., Cruz A.G., Putnik P. (2020). Functional foods: Product development, technological trends, efficacy testing, and safety. Annu. Rev. Food Sci. Technol..

[3] Shaito A., Thuan D.T.B., Phu H.T., Nguyen T.H.D., Hasan H., Halabi S., Abdelhady S., Nasrallah G.K., Eid A.H., Pintus G. (2020). Herbal medicine for cardiovascular diseases: Efficacy, mechanisms, and safety. Front. Pharmacol..

[4] Liu T.T., Liu X.T., Chen Q.X., Shi Y. (2020). Lipase inhibitors for obesity: A review. Biomed. Pharmacother..

[5] Siegień J., Buchholz T., Popowski D., Granica S., Osińska E., Melzig M.F., Czerwińska M.E. (2021). Pancreatic lipase and α-amylase inhibitory activity of extracts from selected plant materials after gastrointestinal digestion in vitro. Food Chem..

[6] Altay M. (2022). Acarbose is again on the stage. World J. Diabetes.

[7] Bhupathiraju S.N., Hu F.B. (2016). Epidemiology of obesity and diabetes and their cardiovascular complications. Circ. Res..

[8] Ortega F.B., Lavie C.J., Blair S.N. (2016). Obesity and cardiovascular disease. Circ. Res..

[9] Chan N., Sobieraj-Teague M., Eikelboom J.W. (2020). Direct oral anticoagulants: Evidence and unresolved issues. Lancet.

[10] Birari R.B., Bhutani K.K. (2007). Pancreatic lipase inhibitors from natural sources: Unexplored potential. Drug Discov. Today.

[11] McDougall G.J., Kulkarni N.N., Stewart D. (2009). Berry polyphenols inhibit pancreatic lipase activity in vitro. Food Chem..

[12] Papoutsis K., Zhang J., Bowyer M.C., Brunton N., Gibney E.R., Lyng J. (2021). Fruit, vegetables, and mushrooms for the preparation of extracts with α-amylase and α-glucosidase inhibition properties: A review. Food Chem..

[13] Bijak M., Sut A., Kosiorek A., Saluk-Bijak J., Golanski J. (2019). Dual anticoagulant/antiplatelet activity of polyphenolic grape seeds extract. Nutrients.

[14] Noad R.L., Rooney C., McCall D., Young I.S., McCance D., McKinley M.C., Woodside J.V., McKeown P.P. (2016). Beneficial effect of a polyphenol-rich diet on cardiovascular risk: A randomised control trial. Heart.

[15] Shah S.B., Sartaj L., Ali F., Shah S.I.A., Khan M.T. (2018). Plant extracts are the potential inhibitors of α-amylase: A review. MOJ Bioequiv. Bioavailab..

[16] Xiao J., Ni X., Kai G., Chen X. (2013). A review on structure–activity relationship of dietary polyphenols inhibiting α-amylase. Crit. Rev. Food Sci. Nutr..

[17] Sosnowska D., Podsędek A., Kucharska A.Z. (2022). Proanthocyanidins as the main pancreatic lipase inhibitors in chokeberry fruits. Food Funct..

[18] Huang R., Zhang Y., Shen S., Zhi Z., Cheng H., Chen S., Ye X. (2020). Antioxidant and pancreatic lipase inhibitory effects of flavonoids from different citrus peel extracts: An in vitro study. Food Chem..

[19] Noorolahi Z., Sahari M.A., Barzegar M., Ahmadi Gavlighi H. (2020). Tannin fraction of pistachio green hull extract with pancreatic lipase inhibitory and antioxidant activity. J. Food Biochem..

[20] Zhang J., Kang M.J., Kim M.J., Kim M.E., Song J.H., Lee Y.M., Kim J.I. (2008). Pancreatic lipase inhibitory activity of *Taraxacum officinale* in vitro and in vivo. Nutr. Res. Pract..

[21] Pukalskienė M., Slapšytė G., Dedonytė V., Lazutka J.R., Mierauskienė J., Venskutonis P.R. (2018). Genotoxicity and antioxidant activity of five *Agrimonia* and *Filipendula* species plant extracts evaluated by comet and micronucleus assays in human lymphocytes and Ames *Salmonella*/microsome test. Food Chem. Toxicol..

[22] Pukalskienė M., Venskutonis P.R., Pukalskas A. (2015). Phytochemical composition and antioxidant properties of *Filipendula vulgaris* as a source of healthy functional ingredients. J. Funct. Foods.

[23] Bijttebier S., Van der Auwera A., Voorspoels S., Noten B., Hermans N., Pieters L., Apers S. (2016). A first step in the quest for the active constituents in *Filipendula ulmaria* (Meadowsweet): Comprehensive phytochemical identification by liquid chromatography coupled to quadrupole-orbitrap mass spectrometry. Planta Medica.

[24] Olennikov D.N., Kruglova M.Y. (2013). A new quercetin glycoside and other phenolic compounds from the genus *Filipendula*. Chem. Nat. Compd..

[25] (2020). European Pharmacopoeia.

[26] Ložienė K., Būdienė J., Vaitiekūnaitė U., Pašakinskienė I. (2023). Variations in Yield, Essential Oil, and Salicylates of *Filipendula ulmaria* Inflorescences at Different Blooming Stages. Plants.

[27] Katanić J., Boroja T., Stanković N., Mihailović V., Mladenović M., Kreft S., Vrvić M.M. (2015). Bioactivity, stability and phenolic characterization of *Filipendula ulmaria* (L.) Maxim. Food Funct..

[28] Akram M., Rashid A. (2017). Anti-coagulant activity of plants: Mini review. J. Throm Thrombolysis.

[29] Agnieszka M., Michał S., Robert K. (2020). Selection of conditions of ultrasound-assisted, three-step extraction of ellagitannins from selected berry fruit of the Rosaceae family using the Response Surface Methodology. Food Anal. Methods.

[30] Harbourne N., Jacquier J.C., O’Riordan D. (2009). Optimisation of the aqueous extraction conditions of phenols from meadowsweet (*Filipendula ulmaria* L.) for incorporation into beverages. Food Chem..

[31] Da Porto A., Cavarape A., Colussi G., Casarsa V., Catena C., Sechi L.A. (2021). Polyphenols rich diets and risk of type 2 diabetes. Nutrients.

[32] Boccellino M., D’Angelo S. (2020). Anti-obesity effects of polyphenol intake: Current status and future possibilities. Int. J. Mol. Sci..

[33] Thomford N.E., Senthebane D.A., Rowe A., Munro D., Seele P., Maroyi A., Dzobo K. (2018). Natural products for drug discovery in the 21st century: Innovations for novel drug discovery. Int. J. Mol. Sci..

[34] Dzobo K. (2022). The role of natural products as sources of therapeutic agents for innovative drug discovery. Compr. Pharmacol..

[35] Olennikov D.N., Kashchenko N.I., Chirikova N.K. (2016). Meadowsweet teas as new functional beverages: Comparative analysis of nutrients, phytochemicals and biological effects of four *Filipendula* species. Molecules.

[36] Gainche M., Ogeron C., Ripoche I., Senejoux F., Cholet J., Decombat C., Delort L., Berthon J.Y., Saunier E., Caldefie Chezet F. (2021). Xanthine oxidase inhibitors from *Filipendula ulmaria* (L.) Maxim. and their efficient detections by HPTLC and HPLC analyses. Molecules.

[37] Katanić J., Pferschy-Wenzig E.M., Mihailović V., Boroja T., Pan S.P., Nikles S., Kretschmer N., Rosić G., Selaković D., Joksimović J. (2018). Phytochemical analysis and anti-inflammatory effects of *Filipendula vulgaris* Moench extracts. Food Chem. Toxicol..

[38] Blazics B., Papp I., Kéry Á. (2010). LC–MS qualitative analysis and simultaneous determination of six *Filipendula* salicylates with two standards. Chromatographia.

[39] Álvarez-Fernández M.A., Hornedo-Ortega R., Cerezo A.B., Troncoso A.M., García-Parrilla M.C. (2014). Effects of the strawberry (*Fragaria ananassa*) purée elaboration process on non-anthocyanin phenolic composition and antioxidant activity. Food Chem..

[40] Garcia-Villalba R., Espín J.C., Aaby K., Alasalvar C., Heinonen M., Jacobs G., Voorspoels S., Koivumäki T., Kroon P.A., Pelvan E. (2015). Validated method for the characterization and quantification of extractable and nonextractable ellagitannins after acid hydrolysis in pomegranate fruits, juices, and extracts. J. Agric. Food Chem..

[41] de Souza L.M., Cipriani T.R., Iacomini M., Gorin P.A., Sassaki G.L. (2008). HPLC/ESI-MS and NMR analysis of flavonoids and tannins in bioactive extract from leaves of *Maytenus ilicifolia*. J. Pharm. Biomed. Anal..

[42] Katanić J., Matić S., Pferschy-Wenzig E.M., Kretschmer N., Boroja T., Mihailović V., Stanković V., Stanković N., Mladenović M., Stanić S. (2017). *Filipendula ulmaria* extracts attenuate cisplatin-induced liver and kidney oxidative stress in rats: In vivo investigation and LC-MS analysis. Food Chem. Toxicol..

[43] Savina T., Lisun V., Feduraev P., Skrypnik L. (2023). Variation in Phenolic Compounds, Antioxidant and Antibacterial Activities of Extracts from Different Plant Organs of Meadowsweet (*Filipendula ulmaria* (L.) Maxim.). Molecules.

[44] Klimczak U., Woźniak M., Tomczyk M., Granica S. (2017). Chemical composition of edible aerial parts of meadow bistort (*Persicaria bistorta* (L.) Samp.). Food Chem..

[45] Saldanha L.L., Vilegas W., Dokkedal A.L. (2013). Characterization of flavonoids and phenolic acids in *Myrcia bella cambess*. Using FIA-ESI-IT-MSn and HPLC-PAD-ESI-IT-MS combined with NMR. Molecules.

[46] de Bont J., Jaganathan S., Dahlquist M., Persson Å., Stafoggia M., Ljungman P. (2022). Ambient air pollution and cardiovascular diseases: An umbrella review of systematic reviews and meta-analyses. J. Intern. Med..

[47] Kudriashov B.A., Liapina L.A., Azieva L.D. (1990). The content of a heparin-like anticoagulant in the flowers of the meadowsweet (*Filipendula ulmaria*). Famakol Toksikol..

[48] Pochet L., Frédérick R., Masereel B. (2004). Coumarin and isocoumarin as serine protease inhibitors. Curr. Pharm. Des..

[49] Bijak M., Bobrowski M., Borowiecka M., Podsędek A., Golański J., Nowak P. (2011). Anticoagulant effect of polyphenols-rich extracts from black chokeberry and grape seeds. Fitoterapia.

[50] Pawlaczyk I., Czerchawski L., Pilecki W., Lamer-Zarawska E., Gancarz R. (2009). Polyphenolic-polysaccharide compounds from selected medicinal plants of *Asteraceae* and *Rosaceae* families: Chemical characterization and blood anticoagulant activity. Carbohydr. Polym..

[51] Seo E.S., Lee J.H., Park J.Y., Kim D., Han H.J., Robyt J.F. (2005). Enzymatic synthesis and anti-coagulant effect of salicin analogs by using the *Leuconostoc mesenteroides* glucansucrase acceptor reaction. J. Biotechnol..

[52] Veličković D., Dimitrijević A., Bihelović F., Bezbradica D., Jankov R., Milosavić N. (2011). A highly efficient diastereoselective synthesis of α-isosalicin by maltase from Saccharomyces cerevisiae. Process Biochem..

[53] Rudeekulthamrong P., Kaulpiboon J. (2016). Application of amylomaltase for the synthesis of salicin-α-glucosides as efficient anticoagulant and anti-inflammatory agents. Carbohydr. Res..

[54] Olennikov D.N., Chemposov V.V., Chirikova N.K. (2021). Metabolites of prickly rose: Chemodiversity and digestive-enzyme-inhibiting potential of *Rosa acicularis* and the main ellagitannin rugosin D. Plants.

[55] Okuda T., Hatano T., Ogawa N. (1982). Rugosin D, E, F and G, dimeric and trimeric hydrolyzable tannins. Chem. Pharm. Bull..

[56] Ochir S., Nishizawa M., Park B.J., Ishii K., Kanazawa T., Funaki M., Yamagishi T. (2010). Inhibitory effects of *Rosa gallica* on the digestive enzymes. J. Nat. Med..

[57] Kato E., Yama M., Nakagomi R., Shibata T., Hosokawa K., Kawabata J. (2012). Substrate-like water soluble lipase inhibitors from *Filipendula kamtschatica*. Bioorg. Med. Chem. Lett..

[58] Singh G.U., Suresh S., Bayineni V.K., Kadeppagari R.K. (2015). Lipase inhibitors from plants and their medical applications. Int. J. Pharm. Pharm. Sci..

[59] Moreno-Córdova E.N., Arvizu-Flores A.A., Valenzuela-Soto E.M., García-Orozco K.D., Wall-Medrano A., Alvarez-Parrilla E., Ayala-Zavala J.F., Domínguez-Avila J.A., González-Aguilar G.A. (2020). Gallotannins are uncompetitive inhibitors of pancreatic lipase activity. Biophys. Chem..

[60] Oliveira R.F., Gonçalves G.A., Inácio F.D., Koehnlein E.A., De Souza C.G.M., Bracht A., Peralta R.M. (2015). lipase. Nutrients.

[61] Aksenov A.A., Krol T.A. Composition and content of phenolic compounds in the leaves of three *Cornus* species. Proceedings of the Modern Trends in the Development of Health Saving Technologies.

[62] Chang C.C., Yang M.H., Wen H.M., Chern J.C. (2002). Estimation of total flavonoid content in propolis by two complementary colorimetric methods. J. Food Drug Anal..

[63] Mammen D., Daniel M. (2012). A critical evaluation on the reliability of two aluminum chloride chelation methods for quantification of flavonoids. Food Chem..

[64] Singleton V.L., Rossi J.A. (1965). Colorimetry of total phenolics with phosphomolybdic-phosphotungstic acid reagents. Am. J. Enol. Vitic..

[65] Ojha S., Raj A., Roy A., Roy S. (2018). Extraction of total phenolics, flavonoids and tannins from *Paederia foetida* L. Leaves and their relation with antioxidant activity. Pharmacogn. J..

[66] Panteghini M., Bonora R., Pagani F. (2001). Measurement of pancreatic lipase activity in serum by a kinetic colorimetric assay using a new chromogenic substrate. Ann. Clin. Biochem..

[67] Morishita Y., Iinuma Y., Nakashima N., Majima K., Mizuguchi K., Kawamura Y. (2000). Total and pancreatic amylase measured with 2-chloro-4-nitrophenyl-4-O-β-D-galactopyranosylmaltoside. Clin. Chem..

